# Roles of palmitoylation and the KIKK membrane-targeting motif in leukemogenesis by oncogenic KRAS4A

**DOI:** 10.1186/s13045-015-0226-1

**Published:** 2015-12-30

**Authors:** Huanbin Zhao, Ping Liu, Ruihong Zhang, Min Wu, Donghe Li, Xuemei Zhao, Chun Zhang, Bo Jiao, Bing Chen, Zhu Chen, Ruibao Ren

**Affiliations:** State Key Laboratory of Medical Genomics, Institute of Health Sciences, Shanghai Institutes for Biological Sciences and Graduate School, Chinese Academy of Sciences, Shanghai Institute of Hematology, Collaborative Innovation Center of Hematology, Ruijin Hospital affiliated to Shanghai Jiao Tong University School of Medicine, 200025 Shanghai, China; State Key Laboratory of Medical Genomics, Shanghai Institute of Hematology, Collaborative Innovation Center of Hematology, Ruijin Hospital affiliated to Shanghai Jiao Tong University School of Medicine, 200025 Shanghai, China; Department of Biology, Brandeis University, Waltham, MA USA

**Keywords:** RAS, Leukemogenesis, Drug target, Plasma membrane translocation, Signal transduction

## Abstract

**Background:**

We have previously shown that palmitoylation is essential for NRAS leukemogenesis, suggesting that targeting RAS palmitoylation may be an effective therapy for NRAS-related cancers. For KRAS-driven cancer, although much research has been focused on the KRAS4B splice variant, which does not undergo palmitoylation, KRAS4A has recently been shown to play an essential role in the development of carcinogen-induced lung cancer in mice and to be widely expressed in human cancers. However, the role of palmitoylation in KRAS4A tumorigenesis is not clear.

**Methods:**

The expression of KRAS4A in *KRAS*-mutated leukemia cell lines and acute myeloid leukemia (AML) cells were checked using western blotting and reverse transcriptions-quantitative polymerase chain reaction (RT-qPCR) analysis, respectively. The leukemogenic potentials of oncogenic KRAS4A and its palmitoylation-defective mutants were examined by a mouse bone marrow transduction and transplantation model and the in vitro transformation assays. The activation of the RAS downstream signaling pathways and the membrane localizations of the KRAS4A and its mutants were analyzed via western blot analysis and confocal microscopy, respectively.

**Results:**

We show here that KRAS4A is expressed in human leukemia cell lines and in AML cells harboring *KRAS* mutations and that mutation at the palmitoylation site of oncogenic KRAS4A significantly abrogates its leukemogenic potential. However, unlike NRAS, palmitoylation-defective KRAS4A still induces leukemia in mice, albeit with a much longer latency. Using NRAS/KRAS4A chimeric constructs, we found that the KIKK motif of KRAS4A contributes to the transforming activity of KRAS4A. Mutations at both palmitoylation site and the KIKK motif abolish the ability of oncogenic KRAS4A to induce leukemia in mice.

**Conclusions:**

Our studies suggest that therapies targeting RAS palmitoylation may also be effective in treating KRAS4A associated malignancies and that interfering the KIKK membrane-targeting motif would enhance the therapeutic effectiveness.

## Background

RAS small GTPases act as molecular binary switches in signal transduction regulating cell proliferation, survival, and differentiation [1]. When bound with GTP, RAS proteins can mediate diverse cellular processes by engaging many effector pathways like RAF-MEK-ERK and PI3K-AKT [2, 3]. Mammalian RAS family includes three *RAS* genes, which encode four highly homologous proteins: HRAS, NRAS, KRAS4A, and KRAS4B. The latter two are alternative splicing isoforms differing only at the carboxyl terminus. These isoforms possess over 90 % identity in the first 166 amino acid residues (G domain, including switch loops and the binding surfaces for downstream effectors) and are mainly diverse in the carboxyl terminal hypervariable region (HVR). Aberrant activation of the RAS signaling pathway is common in cancer, including 20–30 % cancers with *RAS* mutations [4]. Among *RAS* genes, *KRAS* mutations occur most frequently, accounting for 85 % of *RAS* mutations, followed by *NRAS* (12 %) [4]. *HRAS* mutation is relatively rare (3 %) [4].

Despite of intensive research over three decades, cancers harboring *RAS* mutations remain the most difficult to treat and are refractory to current targeted therapies [5]. Though strategies to target oncogenic RAS proteins are emerging, identification of alternative targets that block RAS signaling is critical to develop therapies for RAS-driven cancer [6]. The biological activities of RAS rely on post-translation modifications (PTMs) that target RAS proteins to cell membranes, particularly the plasma membrane [7]. One potential approach to block the RAS oncogenic signaling is, therefore, to inhibit RAS translocation to the plasma membrane. RAS are synthesized as cytosolic proteins. To translocate to membranes, they need first to be modified by prenylation at the cysteine of the carboxyl terminal CAAX motif by farnesyltransferases (FTase) or geranylgeranyltransferase (GGTase), followed by -AAX proteolysis by RAS converting enzyme (RCE) and methylation of the exposed, farnesylated cysteine residue by isoprenylcysteine carboxyl methyltransferase (Icmt) [8]. CAAX motif is the C-terminal tetrapeptide sequence of RAS proteins (C for cysteine, A for aliphatic amino acid, and X for serine or methionine). Since prenylation of RAS by FTase is the obligate step in RAS PTMs, much emphasis had been placed on developing therapies targeting RAS farnesylation, but successes are modest to date due to a redundancy of the FTase and GGTase [9]. Inhibitors targeting both FTase and GGTase in combination have been proved too toxic to be clinically useful [10, 11].

The prenylation of RAS proteins provides the minimal signal for their membrane association. NRAS, HRAS, and KRAS4A are further palmitoylated by palmitoylacyltransferases (PAT) at the cysteine residue(s) upstream of the CAAX motif [12–14]. On the other hand, KRAS4B, which lacks of cysteine residues at its C terminus to accept palmitoylation modification, traffics directly to the plasma membrane (PM) by associating its positively charged polylysine residues in HVR with the negatively charged component of the inner membrane through electrostatic interaction [15, 16].

We have previously shown that palmitoylation is essential for NRAS leukemogenesis, suggesting that targeting RAS palmitoylation may be an effective therapy for NRAS-related cancers [17]. For cancers with KRAS mutations, much research has been focused on KRAS4B, since *KRAS4B* transcript was shown to be more abundant [18]. However, since most oncogenic mutations occur in the G domain of RAS, which is identical for KRAS4A and KRAS4B, KRAS4A should be activated in cancers harboring *KRAS* mutations. Although KRAS4A is dispensable for mouse development [19], accumulating evidences indicate that the altered *KRAS4A/4B* ratios may correlate with progression of lung and colorectal adenocarcinoma [20, 21] and that KRAS4A plays an important role in lung carcinogenesis [22]. Furthermore, a recent study by improved quantitative RT-PCR revealed that the *KRAS4A* splice variant is widely expressed in human cancers [23]. Both KRAS isoforms, therefore, should be taken into account in developing effective cancer therapies.

The role of palmitoylation in KRAS4A tumorigenesis in vivo is not known. In this study, we compared the effect of palmitoylation on signaling and leukemogenic potential of oncogenic NRAS and KRAS4A. We found that palmitoylation also plays a critical role in KRAS4A leukemogenesis, but KRAS4A contains an additional membrane association motif that contributes to the oncogenic activity of KRAS4A in vivo.

## Results

### KRAS4A is expressed in human hematologic malignant cells with *KRAS* mutations

**Fig. 1:**
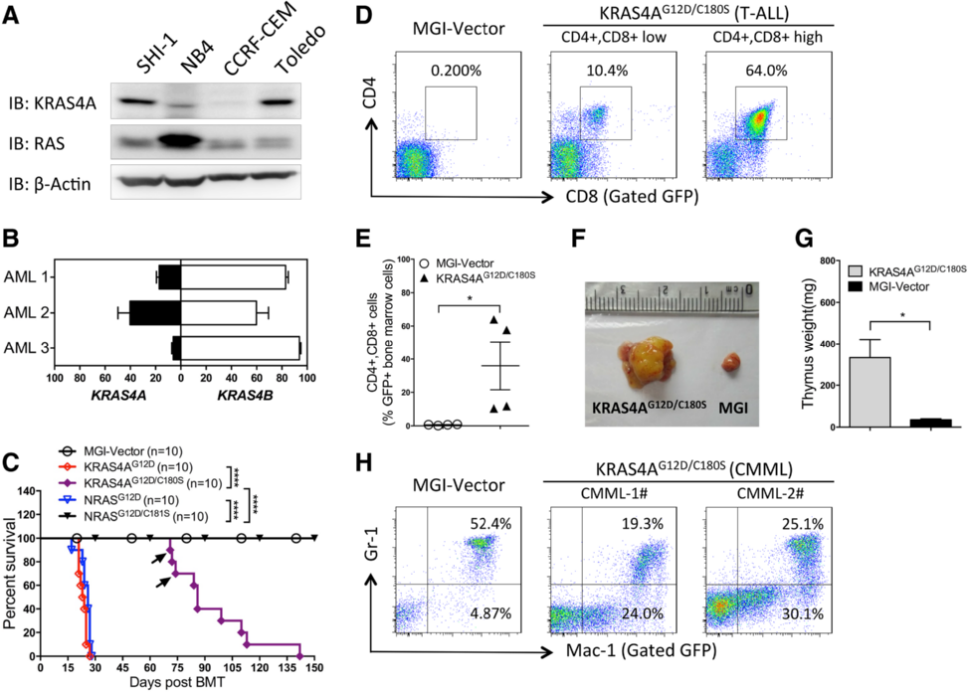
Effects of palmitoylation on leukemogenesis of oncogenic NRAS vs. KRAS4A. **a** The expression levels of KRAS4A in *KRAS*-mutated leukemia cell lines indicated were detected by western blot analysis using an anti-KRAS4A specific antibody. Total RAS and actin were detected as controls. **b** Quantification of the relative abundance of *KRAS4A* and *KRAS4B* slice variants in bone marrow cells from AML patients with oncogenic *KRAS* mutations. The results are presented as means ± SD. **c** Kaplan-Meier plot of cumulative survival of recipient mice transplanted with bone marrow cells infected by KRAS4A^G12D^, KRAS4A^G12D/C180S^, NRAS^G12D^, or NRAS^G12D/C181S^ containing retroviruses. The observation period was over 1 year after BMT. The median survival is 23, 26, and 86 days for KRAS4A^G12D^, NRAS^G12D^, and KRAS4A^G12D/C180S^, respectively. Disease incidences: KRAS4A^G12D^ (9/10 CMML; 1/10 AML), KRAS4A^G12D/C180S^ (2/10 CMML; 8/10 T-ALL), NRAS^G12D^ (5/10 CMML; 5/10 AML). *Arrows* represent the two moribund KRAS4A^G12D/C180S^ mice diagnosed as CMML-like disease. *P* values were determined by the log-rank test. ****P < 0.0001; *n* = 10 in each group. **d** Representative analyses of leukemic bone marrow cells from the moribund KRAS4A^G12D/C180S^ mice with T-ALL like disease (8 out of 10) by flow cytometry are shown. The MGI vector mice were used as control. **e** Comparison of CD4^+^, CD8^+^ cell percentages in GFP+ BM cells from the moribund KRAS4A^G12D/C180S^ mice with T-ALL like disease (*n* = 4) and the MGI vector mice (*n* = 4). Student *t* test was used for the statistical analysis. *P < 0.05. **f** Representative thymus from moribund KRAS4A^G12D/C180S^ mice with T-ALL like disease and that from the MGI vector mice. **g** Comparison of weight of thymus from moribund KRAS4A^G12D/C180S^ mice with T-ALL like disease vs. that from MGI vector mice. The data were presented as mean ± SEM; *P < 0.05; *n* = 3 in each group. **h** Representative analyses of leukemic bone marrow cells from moribund KRAS4A^G12D/C180S^ mice with CMML-like disease (2 out of 10). The MGI vector mice were used as control

*KRAS* mutations have been found in hematologic malignancies [24]. To determine whether KRAS4A is expressed in blood cancer cells harboring *KRAS* mutations, we checked the KRAS4A protein levels with a KRAS4A specific antibody in acute myeloid leukemia cell lines SHI-1 and NB4, T cell acute lymphoblastic leukemia cell line CCRF-CEM, as well as diffuse large B cell lymphoma cell line Toledo. All of these four cell lines derived from human hematologic malignancies with *KRAS* mutations. We observed that KRAS4A is expressed at various levels in these cell lines, with a particularly high level in SHI-1 and Toledo cells (Fig. 1a).

To confirm our observation in the hematologic malignant cells from patients, we examined the relative abundance of *KRAS4A* and *KRAS4B* mRNA in bone marrow samples from patients suffering acute myeloid leukemia (AML) with oncogenic *KRAS* mutations by reverse transcriptions-quantitative polymerase chain reaction (RT-qPCR) as previously described [23]. We found that *KRAS4A* accounts for 6.08–40.21 % of total *KRAS* transcripts in these samples (Fig. 1b). The results demonstrate that KRAS4A is expressed in hematologic malignant cells with *KRAS* mutations.

### Palmitoylation is critical but not essential for the leukemogenesis by oncogenic KRAS4A

We have previously shown that both oncogenic NRAS and KRAS4B can induce myeloid leukemia in a mouse bone marrow transduction/transplantation (BMTT) model [25, 26]. Here, we checked whether KRAS4A with the oncogenic mutation G12D (KRAS4A^G12D^) could also induce leukemia in mice and what is the role of palmitoylation if KRAS4A^G12D^ does have leukemogenic potential. We constructed retroviral vectors expressing Myc-tagged KRAS4A^G12D^ and its palmitoylation-defective mutant KRAS4A^G12D/C180S^. We infected BM cells isolated from 5-fluorouracil-treated mice with titer-matched retroviruses containing KRAS4A^G12D^ or KRAS4A^G12D/C180S^ and then transplanted these cells into lethally irradiated syngeneic recipient mice, as previously described [26]. As controls, mice receiving NRAS^G12D^, NRAS^G12D/C181S^, or the MGI vector-transduced bone marrow cells were generated. We found that KRAS4A^G12D^ mice succumbed to disease in 20 to 40 days post bone marrow transplantation (BMT), with similar disease latency as NRAS^G12D^ (Fig. 1c). Mice receiving KRAS4A^G12D/C180S^-transduced bone marrow cells also died in succession but with a much longer disease latency compared with KRAS4A^G12D^ (Fig. 1c). In contrast, all mice receiving MGI vector or NRAS^G12D/C181S^-transduced bone marrow cells remained healthy for over one year (Fig. 1c and data not shown).

We sacrificed the moribund mice receiving KRAS4A^G12D^, KRAS4A^G12D/C180S^, and NRAS^G12D^-transduced bone marrow cells to examine their disease phenotype. Analysis of peripheral blood, bone marrow, livers, and spleens revealed that KRAS4A^G12D^ mice developed chronic myelomonocytic leukemia (CMML)- or AML-like diseases similar to NRAS^G12D^ mice as previously shown [25, 26] (data not shown). All KRAS4A^G12D/C180S^ mice developed leukocytosis, anemia, and enlarged spleens and livers, in spite of the normal phenotype in the first analysis at 3 weeks post BMT (data not shown). Fluorescence-activated cell sorting (FACS) analyses revealed that 8 out of 10 KRAS4A^G12D/C180S^ mice developed T cell acute lymphoblastic leukemia (T-ALL) like disease (Fig. 1d, e). Thymus is enlarged in these mice (Fig. 1f, g). The rest two KRAS4A^G12D/C180S^ mice developed a CMML-like disease (Fig. 1h and data not shown).

These results demonstrate that like NRAS, HRAS, and KRAS4B, oncogenic KRAS4A also possesses leukemogenic activity, though it induces mostly T-ALL like disease rather than myeloid leukemia when palmitoylation is blocked. The difference in cellular localization between different RAS isoforms may account for the distinguished leukemia phenotypes. The data also show that though palmitoylation plays an important role in KRAS4A leukemogenesis, unlike NRAS, it is not essential.

### Differential roles of palmitoylation on transformation and signal transduction by oncogenic NRAS and KRAS4A

**Fig. 2:**
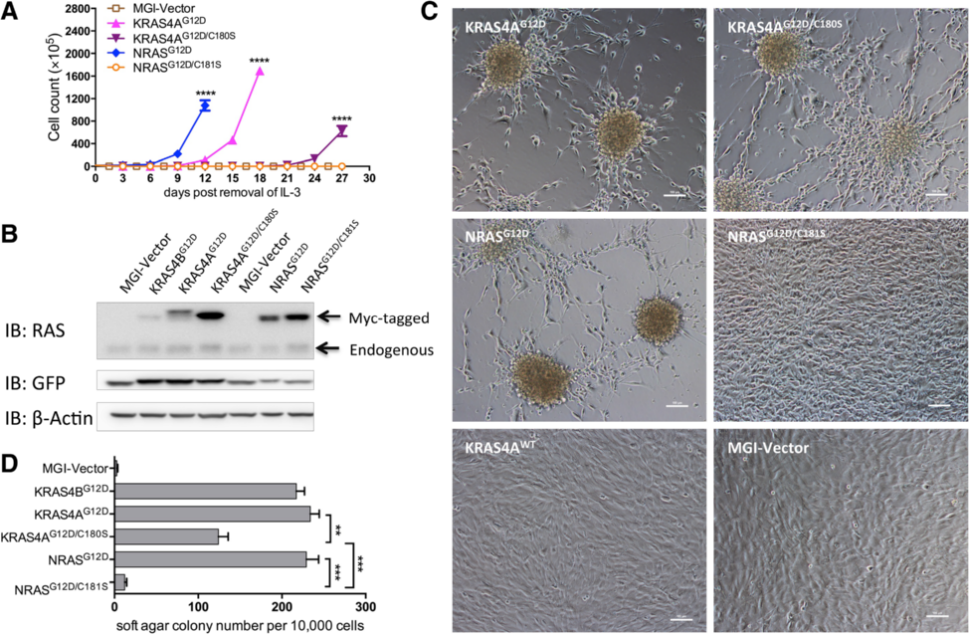
Effects of palmitoylation on transformation potential of oncogenic NRAS vs. KRAS4A in vitro. **a** The growth curves of Ba/F3 cells transduced with the MGI vector control, KRAS4A^G12D^, KRAS4A^G12D/C180S^, NRAS^G12D^, or NRAS^G12D/C181S^ after removal of IL-3. Student *t* test was used for the statistical analysis of the cell numbers in triplicate at each time point. ****P < 0.0001. **b** Immunoblotting of lysates of NIH3T3 cells stably expressing the GFP control, KRAS4A^G12D^, KRAS4A^G12D/C180S^, NRAS^G12D^, or NRAS^G12D/C181S^ using a pan anti-RAS antibody. The *top band* represents Myc-tagged-RAS; the *bottom band* represents endogenous RAS. **c** Morphology of cultured NIH3T3 cells stably expressing the indicated proteins was observed under light microscope (original magnification, ×200). **d** Quantification of anchorage-independent growth of NIH3T3 cells expressing the indicated proteins. Colonies were counted on day 14 after plating in triplicate. Student *t* test was used for the statistical analysis. **P < 0.01, ***P < 0.001

To further compare the effect of palmitoylation defectiveness on the oncogenic potential of KRAS4A and NRAS, we infected the Ba/F3 cell line, an IL-3 dependent mouse hematopoietic progenitor cell line, with retroviruses containing green fluorescent protein (GFP) alone (MGI vector control), KRAS4A^G12D^, KRAS4A^G12D/C180S^, NRAS^G12D^, or NRAS^G12D/C181S^, and sorted for GFP-positive cells to generate corresponding cell lines. After removal of IL-3 from the culture medium, each group of cells was cultured and counted every three days in triplicate. Ba/F3 cells stably expressing GFP alone (MGI vector) stopped growing and died within first 3 days due to the lack of stimulation by IL-3, whereas cells expressing NRAS^G12D^ and KRAS4A^G12D^ continued to proliferation after removal of IL-3 (Fig. 2a). Cells expressing KRAS4A^G12D/C180S^ displayed a significantly slower proliferation rate compared with KRAS4A. NRAS^G12D/C181S^, on the other hand, is incapable of rendering Ba/F3 cells IL-3 independent growth (Fig. 2a). Overall, the in vitro transforming activities of the oncogenic NRAS, KRAS4A, and their palmitoylation mutants correlate with their leukemogenic potentials in vivo.

We previously showed that palmitoylation-deficient NRAS^G12D^ confers morphologic changes, abrogates cell density-dependent inhibition of growth but does not confer anchorage-independent growth to NIH3T3 cells [17]. To determine the effect of palmitoylation on transformation of NIH3T3 cells by oncogenic KRAS4A, we infected NIH3T3 cells with KRAS4A^G12D^ and KRAS4A^G12D/C180S^ retroviruses. NRAS^G12D^, NRAS^G12D/C181S^, KRAS4B^G12D^, and the MGI vector were included as controls. To rule out the possibility whether the myc tag could modify tumorigenic activity of oncogenic RAS, we also infected NIH3T3 cells with myc-tagged wild type KRAS4A retrovirus and found that the myc tag does not alter the biological activity of wild type KRAS4A in this assay (Fig. 2c). RAS protein expression levels in NIH3T3 were detected by western blot analysis (Fig. 2b). It is known that high levels of RAS can induce cellular senescence [27]. The low expression level of oncogenic KRAS4B, hence, may be due to the negative selection of cells with high expression of KRAS4B^G12D^.

We found that similar to the morphology of NIH3T3 cells expressing NRAS^G12D^, cells expressing KRAS4A^G12D^ and KRAS4A^G12D/C180S^ both appeared smaller and more spindle-shaped than the NRAS^G12D/C181S^ and MGI control cells and began to grow into spheres from the nodules in a lattice (Fig. 2c), which resemble the cancer stem cells (CSCs)-like properties ([28] and unpublished data by Frank McCormick and colleagues). We further assessed the effect of palmitoylation deficiency on the transforming potential of KRAS4A^G12D^ by soft agar colony assay. NIH3T3 cells expressing KRAS4A^G12D/C180S^ generated fewer colonies than KRAS4A^G12D^ but significantly more colonies than NRAS^G12D/C181S^ (Fig. 2d). These results also correlate in general with the leukemogenic potentials of NRAS, KRAS4A, and their palmitoylation mutants in vivo.

As the in vitro transforming activities of NRAS, KRAS4A, and their palmitoylation mutants in NIH3T3 cells correlate well with their transforming activities in Ba/F3 cells as well as their in vivo leukemogenic potentials, we went on to check the effect of palmitoylation on NRAS and KRAS4A signaling using the NIH3T3 cell lines expressing RAS variants described above. It is well known that the transforming potential of oncogenic RAS relies on multiple downstream signaling pathways, including the RAS/RAF/MEK/ERK and PI3K/AKT pathways [1, 2, 29]. We examined known activating phosphorylation sites of well-identified signaling proteins in the RAS/RAF/MEK/ERK and PI3K/AKT pathways in KRAS4A^G12D^-, KRAS4A^G12D/C180S^-, NRAS^G12D^-, or NRAS^G12D/C181S^-expressing NIH3T3 cells by Western blotting analysis using specific phospho-protein antibodies. NIH3T3 cells expressing GFP alone or KRAS4B^G12D^ were included as controls. Cells were serum-starved for 22 h before lysis to minimize RAS signaling background activated by serum growth factors.

**Fig. 3:**
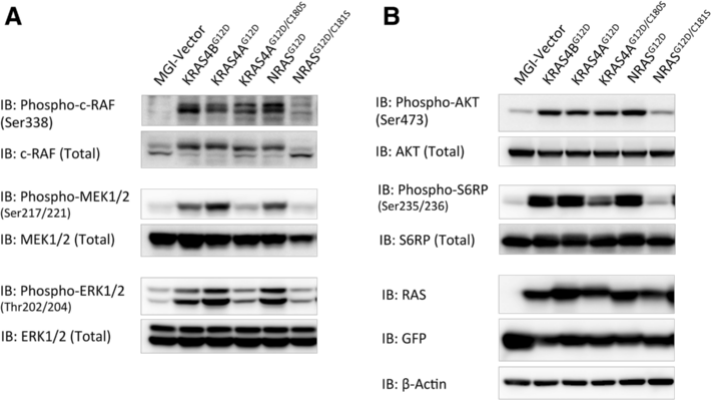
Effects of palmitoylation on signaling transduction of oncogenic NRAS vs. KRAS4A. Serum-starved lysates of NIH3T3 cells expressing GFP, KRAS4A^G12D^, KRAS4A^G12D/C180S^, NRAS^G12D^, or NRAS^G12D/C181S^ were analyzed by western blotting. **a** Phospho-c-RAF, MEK, and ERK1/2 antibodies were used to detect the effects of palmitoylation deficiency on the MEK-ERK signaling pathway. **b** Phospho-AKT and S6RP antibodies were used to detect the effects of palmitoylation deficiency on the PI3K-AKT signaling pathway. Expression levels of RAS, GFP, and β-Actin were detected as loading controls

As expected, the RAF/MEK/ERK pathway is activated by oncogenic KRAS4B, KRAS4A, and NRAS (Fig. 3a). Interestingly, palmitoylation deficiency greatly abrogates the ability of NRAS^G12D^, but not KRAS4A^G12D^, on activating c-RAF (Fig. 3a). On the other hand, palmitoylation deficiency markedly reduces the ability of both NRAS^G12D^ and KRAS4A^G12D^ in activating MEK1/2 and ERK1/2 (Fig. 3a). The results confirm that activation of MEK/ERK requires conditions other than c-RAF activation [30, 31]. It is also known that c-RAF possesses biological activities other than that through MEK/ERK activation [32]. The above results suggest that in addition to inhibition of the MEK/ERK signaling, completely blocking RAS transformation may involve downstream effectors of c-RAF other than MEK and ERK.

Also as expected, AKT and S6RP are activated by oncogenic KRAS4B, KRAS4A, and NRAS (Fig. 3b). Similar to its effect on c-RAF activation, palmitoylation deficiency greatly abrogates the ability of NRAS^G12D^, but not KRAS4A^G12D^, on activating AKT (Fig. 3b). Palmitoylation deficiency also abrogates the ability of NRAS^G12D^ and, to a lesser extent, of KRAS4A^G12D^, to activate S6RP (Fig. 3b).

The results presented here suggest that palmitoylation deficiency differentially impinges upon signaling pathways of NRAS^G12D^ and KRAS4A^G12D^, which correlates well with their in vitro and in vivo transforming activities and that abolishing RAS oncogenic activity may involve blocking its ability to activate c-RAF, MEK, ERK, AKT, and S6RP.

### The KIKK polybasic motif in HVR of KRAS4A contributes to the oncogenic activity of KRAS4A

**Fig. 4:**
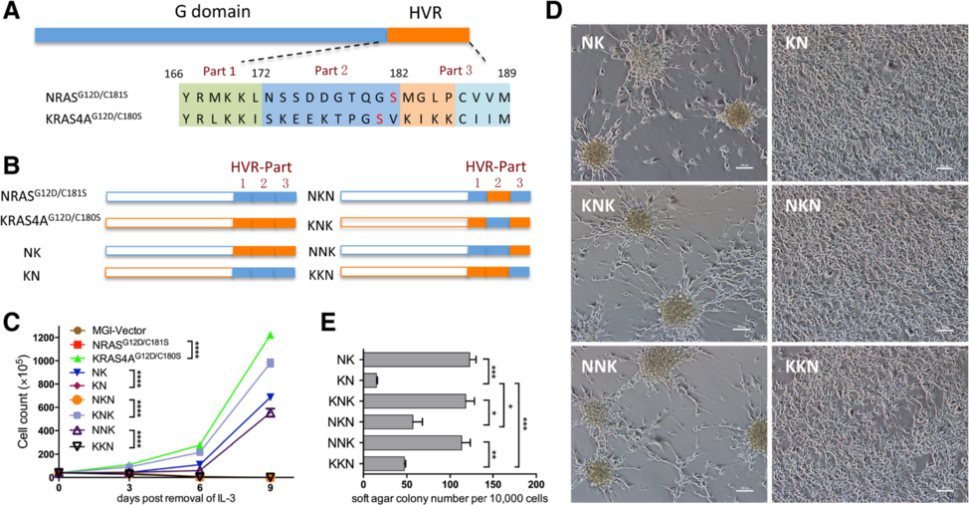
Identification of sequences rendering palmitoylation defective oncogenic KRAS4A transformation active. **a** The sequence alignment of the hypervariable regions (HVRs) of NRAS vs. KRAS4A. The *shaded areas* in *green*, *blue*, or *orange* indicate the sequences separated according to the lysine enrichment. Part 1: amino acids 166–172; part 2: amino acids 173–182; part 3: amino acids 183–189. **b** Diagram of NRAS^G12D/C181S^ and KRAS4A^G12D/C180S^ C-terminal domain chimeras. The total C-terminal domain, part 2 or part 3 was swapped respectively to generate NK, KN, NKN, KNK, NNK, and KKN. **c** Growth curves of Ba/F3 cells overexpressing the indicated proteins after removal of IL-3. Student *t* test was used for the statistical analysis of the cell numbers in each timepoint. ****P < 0.001. **d** Morphology of cultured NIH3T3 cells stably expressing the indicated proteins was observed under light microscope (original magnification, ×200). **e** Quantification of anchorage-independent growth of NIH3T3 cells expressing the indicated proteins. Colonies were counted on day 14 after plating in triplicate. Student t test was used for the statistical analysis. *P < 0.05, **P < 0.01, ***P < 0.001

To determine which amino acids contribute to the differential role of palmitoylation in the oncogenesis of KRAS4A and NRAS, the HVR sequence of KRAS4A^G12D/C180S^ and NRAS^G12D/C181S^ (Fig. 4a) was swapped to generate KRAS4A/NRAS chimeric genes named NK and KN (Fig. 4b). We then ectopically expressed these two chimeric genes in Ba/F3 and NIH3T3 cell lines. As controls, cells expressing KRAS4A^G12D/C180S^, NRAS^G12D/C181S^, or GFP alone were generated. After removal of IL-3, Ba/F3 cells expressing NK continued to proliferate, whereas Ba/F3 expressing KN stopped growing and died soon (Fig. 4c). Similarly, NIH3T3 cells expressing NK grew into spheres in a lattice as previously described, whereas NIH3T3 cells expressing KN had a slight morphology change but did not form any sphere (Fig. 4d). Soft agar colony assay also shows that cells expressing NK generate significantly greater number of colonies than that of KN (Fig. 4e). These results indicate that the HVR domain of KRAS4A render KRAS4A^G12D/C180S^ a higher transforming activity than NRAS^G12D/C181S^.

It was reported that HVR of KRAS4A contains a bipartite polybasic region, designated as PB1 (RLKK, aa167-170) and PB2 (KIKK, aa182-185), which can independently deliver KRAS4A to the plasma membrane [23]. Sequence comparison shows that PB1 is also contained in NRAS HVR sequence (Fig. 4a). To further clarify which amino acids in the HVR sequence contribute to the differential effect of palmitoylation defectiveness on oncogenesis of KRAS4A and NRAS, we divided HVR of KRAS4A into three parts: aa166-171, aa172-181, and aa182-189 (part 1, 2, 3 respectively; Fig. 4a, b). We swapped the sequence of aa172-181 and aa182-189 of KRAS4A^G12D/C180S^ and NRAS^G12D/C181S^, respectively, and constructed the corresponding retroviral vectors expressing these chimeric RAS genes named NKN, KNK, NNK, or KKN (Fig. 4b). We then expressed these genes in Ba/F3 and NIH3T3 cell lines. We found that Ba/F3 cells expressing KNK or NNK continue to proliferate after removal of IL-3, whereas Ba/F3 cells expressing NKN or KKN stop growing and die in the absence of IL-3 (Fig. 4c). Consistently, NIH3T3 cells expressing KNK or NNK formed spheres as previously described, whereas cells expressing KNN or NKN did not (Fig. 4d). Soft agar colony assay shows that NIH3T3 cells expressing KNK or NNK generate significant greater numbers of colonies compared with those expressing KNN or NKN, indicating that chimeras containing aa182-189 of KRAS4A possesses higher transforming activity (Fig. 4e). Interestingly, we found that the colony number of KKN or NKN expressing cells was significantly more than KNN or NRAS^G12D/C181S^, suggesting that sequence aa172-181 contributes to the transforming potential of KRAS4A as well (Fig. 4e). These results indicate that the sequence aa182-189 mainly accounts for the differential role of palmitoylation on the oncogenesis of KRAS4A and NRAS.

Amino acids between position 182 and 189 consist of two parts: the KIKK polybasic motif (PB2) and the CAAX box, the latter of which is conserved in all RAS proteins and undergoes prenylation, -AAX proteolysis, and methylation. Hence, the data indicate that the KIKK polybasic motif in HVR of KRAS4A contributes to the differential role of palmitoylation in the oncogenesis of KRAS4A and NRAS.

### The role of the KIKK motif on cellular localization of KRAS4A

**Fig. 5:**
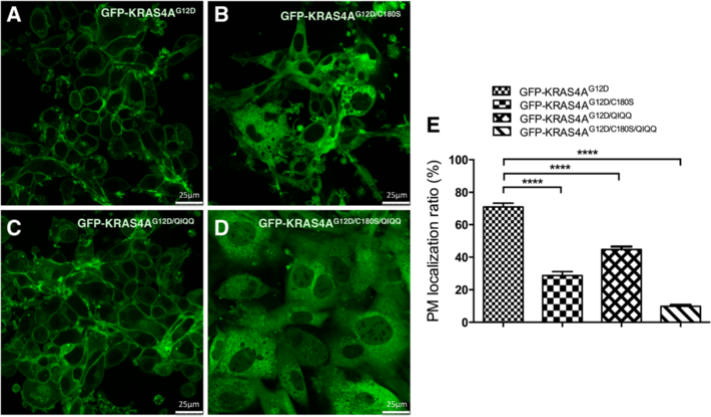
KIKK of KRAS4A functions as a second plasma membrane association motif. NIH3T3 cells expressing GFP-fused KRAS4A^G12D^ **a**, KRAS4A^G12D/C180S^ **b**, KRAS4A^G12D/QIQQ^ **c**, or KRAS4A^G12D/C180S/QIQQ^ **d** were visualized on a Leica TCS 7300 Spectral Confocal Microscope (original magnification, ×630). **e** The comparison of plasma membrane localization ratios of each KRAS4A mutant. Ten representative cells of each group were photographed and analyzed by LAS AF Lite Software. *PM* plasma membrane; Student *t* test was used for the statistical analysis. ****P < 0.0001

To reveal the function of KIKK polybasic motif on KRAS4A plasma membrane association, we expressed N-terminal GFP-fusion KRAS4A^G12D^, KRAS4A^G12D/C180S^, KRAS4A^G12D/QIQQ^ (substitution of lysine residuals in KIKK with glutamines), and KRAS4A^G12D/C180S/QIQQ^ in NIH3T3 cells and checked the protein membrane localization by confocal microscope. A clear and intensive localization of KRAS4A^G12D^ on the plasma membrane of NIH3T3 was observed, whereas nonpalmitoylated KRAS4A^G12D/C180S^ localized mainly on the endomembrane, with only a small fraction of KRAS4A^G12D/C180S^ proteins localize on the plasma membrane, particular at some focal areas (Fig. 5a, b, e). KRAS4A^G12D/QIQQ^, on the other hand, mainly localizes on the plasma membrane, with a slight increase of localization on endomembranes (Fig. 5c, e). The plasma membrane localization of KRAS4A is completely abolished by mutating both the palmitoylation site and KIKK motif (Fig. 5d, e). These results indicate that palmitoylation plays a major role in translocating KRAS4A proteins from endomembranes to the plasma membrane. The KIKK polybasic motif only plays a minor role in translocating KRAS4A from endomembranes to the plasma membrane; it may help the protein to localize on special domains of the plasma membrane, allowing palmitoylation-deficient KRAS4A^G12D^ signals distinctively from palmitoylation-deficient NRAS^G12D^ (Fig. 3).

### The plasma membrane-targeting function of KIKK motif depends on its positively charged residuals

**Fig. 6:**
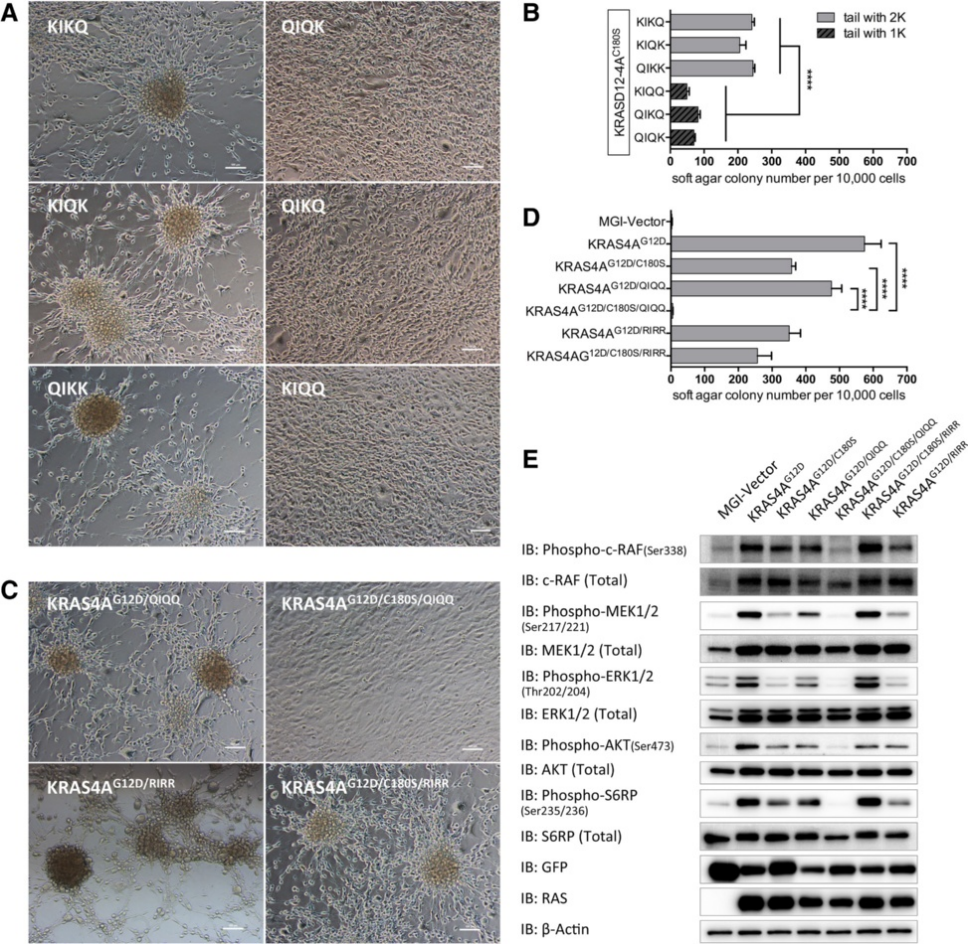
KIKK motif of KRAS4A functions by providing a positive charged region. **a** Morphology of cultured NIH3T3 cells stably expressing KRAS4A^G12D/C180S^ with KIKK motif mutated to QIKK, KIQK, KIKQ or QIQK, QIKQ, KIQQ was observed under light microscope (original magnification, ×200). **b**, **d** Quantification of anchorage-independent growth of NIH3T3 cells expressing the indicated proteins. Colonies were counted on day 14 after plating in triplicate. Student *t* test was used for the statistical analysis. ****P < 0.0001. **c** Morphology of cultured NIH3T3 cells stably expressing KRAS4A^G12D^, KRAS4A^G12D/C180S^, KRAS4A^G12D/QIQQ^, or KRAS4A^G12D/C180S/QIQQ^ was observed under light microscope (original magnification, ×200). **e** Serum-starved lysates of NIH3T3 cells expressing the indicated proteins were analyzed by western blotting to detect the phosphorylation levels of c-RAF, MEK, ERK1/2, AKT, and S6RP. Expressions of RAS, GFP, and β-actin were used as loading controls

To determine whether the KIKK motif of KRAS4A functions by purely providing electrostatic interaction with membrane through its basic amino acids or by unknown interactions, we substituted the lysine residuals in the KIKK motif of KRAS4A^G12D/C180S^ with one or two uncharged glutamine residuals (named QIKK, KIQK, KIKQ, QIQK, QIKQ, and KIQQ, respectively) and overexpressed the mutated genes in NIH3T3 cells. We observed that NIH3T3 cells expressing QIKK, KIQK, or KIKQ formed spheres in a lattice and had other morphology close to KRAS4A^G12D/C180S^, whereas cells expressing QIQK, QIKQ, or KIQQ had spindle-shaped morphology but did not form any sphere (Fig. 6a). Soft agar colony assay also showed a gradual decline in the colony numbers with the decrease of lysine residual numbers in the KIKK motif (Fig. 6b). Meanwhile, no obvious changes in the sphere morphology and the transforming potential were observed after substitution of the lysine residuals in the KIKK motif with arginine residuals (named KRAS4A^G12D/RIRR^ and KRAS4A^G12D/C180S/RIRR^, Fig. 6c, d). These results indicate that the KIKK motif functions by providing electrostatic interaction with the plasma membrane through its positively charged amino acids.

To check the effect of KIKK mutations on signaling of KRAS4A, we infected NIH3T3 cells with retroviral constructs expressing KRAS4A^G12D^, KRAS4A^G12D/C180S^, KRAS4A^G12D/QIQQ^, or KRAS4A^G12D/C180S/QIQQ^. Cells were starved for 22 h and then lysed for detection of the activation of signaling proteins c-RAF, MEK1/2, ERK1/2, AKT, and S6RP. We observed that in KRAS4A^G12D/QIQQ^ expressing cells, the phosphorylation levels of MEK1/2, ERK1/2, AKT, and S6RP were lower than KRAS4A^G12D^ but a slightly higher than KRAS4A^G12D/C180S^ (Fig. 6e). No obvious change in the phosphorylation level of c-RAF Ser338 in KRAS4A^G12D/QIQQ^ cells was observed compared with that of KRAS4A^G12D^ and KRAS4A^G12D/C180S^. Interestingly, the ability of KRAS4A^G12D^ to active c-RAF, MEK1/2, ERK1/2, AKT, and S6RP is abolished by mutating both the palmitoylation site and the KIKK motif (KRAS4A^G12D/C180S/QIQQ^). In contrast, substitution of the lysine residuals with arginine residuals in the KIKK motif does not significantly affect KRAS4A^G12D^ signaling activity. These results further support the conclusion that the KIKK polybasic motif in HVR of KRAS4A contributes to the differential role of palmitoylation in the oncogenic signaling of KRAS4A and NRAS.

### The relative importance of palmitoylation and the KIKK motif on leukemogenic potential of KRAS4A

**Fig. 7:**
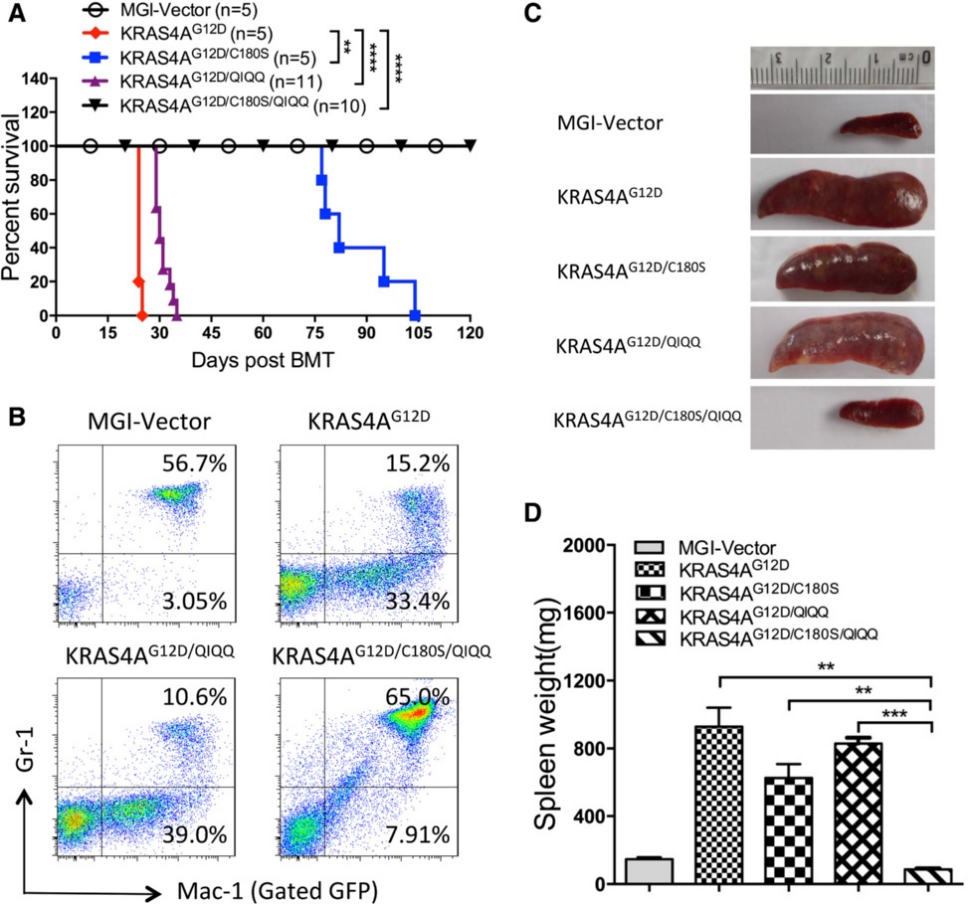
Contributions of palmitoylation and KIKK motif on leukemogenic potential of oncogenic KRAS4A. **a** Kaplan-Meier plot of cumulative survival of recipient mice transplanted with bone marrow cells infected by KRAS4A^G12D^ (*n* = 5), KRAS4A^G12D/C180S^ (*n* = 5), KRAS4A^G12D/QIQQ^ (*n* = 11), KRAS4A^G12D/C180S/QIQQ^ (*n* = 10) containing retroviruses. The MGI vector mice (*n* = 5) were observed as control. *P* values were determined by the log-rank test. **P < 0.01, ****P < 0.0001. **b** Immunophenotyping of the leukemic bone marrow cells isolated from the KRAS4A^G12D^, KRAS4A^G12D/QIQQ^, and KRAS4A^G12D/C180S/QIQQ^ moribund mice by flow cytometry. The myeloid lineage markers include Gr-1 and Mac-1. All the cells are GFP positive gated. **c** Spleens from the mice of each group were shown as indicated. **d** Comparison of the spleen weights of each group mice. The data were presented as mean ± SEM. Student *t* test was used for the statistical analysis. **P < 0.01, ***P < 0.001; *n* = 3 in each group

To further examine the role of KIKK motif in KRAS4A leukemogenesis, we used the BMTT model with retroviruses containing KRAS4A^G12D/QIQQ^ or KRAS4A^G12D/C180S/QIQQ^. Mice receiving KRAS4A^G12D^, KRAS4A^G12D/C180S^, or MGI vector-transduced bone marrow cells were used as controls. As previously shown, KRAS4A^G12D^ rapidly and efficiently induces a CMML- or AML-like disease in mice. Under the same experimental conditions, the disease latency of KRAS4A^G12D/QIQQ^ was only slightly prolonged compared to KRAS4A^G12D^, while KRAS4A^G12D/C180S^ mice survived a much longer time (Fig. 7a). In contrast, all KRAS4A^G12D/C180S/QIQQ^ mice remained healthy during the course of experiment (Fig. 7a). Immunophenotype analysis showed that mice transfected KRAS4A^G12D/QIQQ^ also developed CMML- or AML-like disease (Fig. 7b), while the majority of KRAS4A^G12D/C180S^ mice developed T-ALL like disease as previously described (Fig. 1d and data not shown). All groups of mice in moribund or over 1 year were sacrificed for pathological analysis. The spleens of KRAS4A^G12D/C180S/QIQQ^ mice are normal compared with KRAS4A^G12D^, KRAS4A^G12D/QIQQ^, or KRAS4A^G12D/C180S^ (Fig. 7c). These results indicate that palmitoylation plays a more important role than the KIKK plasma membrane-targeting motif in KRAS4A^G12D^ leukemogenesis, yet blocking both could completely abolish the ability of oncogenic KRAS4A to induce leukemia.

## Discussion

Accumulating evidence has indicated that the *KRAS4A* splice variant plays an important role in tumorigenesis [20–23]. Understanding the oncogenic potential of KRAS4A in vivo and the ways to block its oncogenic signaling is important for developing effective cancer therapies. Here, we show that KRAS4A has a similar leukemogenic potential as NRAS (Fig. 1), as well as KRAS4B (data not shown), when expressed under the same promoter. Palmitoylation is very important for KRAS4A leukemogenesis, but it is not essential as that in case of NRAS. The KIKK motif in KRAS4A functions as an alternative membrane-targeting motif and makes a contribution to leukemogenesis by oncogenic KRAS4A.

Consistent with the results in our previous work, NRAS^G12D^ palmitoylation mutant completely loses the capacity to activate MEK-ERK and PI3K-AKT pathways in NIH3T3 cells [17]. Unlike NRAS^G12D^, although defective in activating MEK, ERK, and S6RP, the KRAS4A^G12D^ palmitoylation mutant retains most of its ability to activate c-RAF and AKT, coincident with the sphere morphology and the more colony numbers of KRAS4A^G12D/C180S^-expressing NIH3T3 cells compared with NRAS^G12D/C181S^-expressing cells. These data suggest that though the plasma membrane localization of KRAS4A^G12D^ is greatly reduced by the palmitoylation mutation (Fig. 5), the KIKK motif may target KRAS4A to a special domain in the plasma membrane, allowing it to activate c-RAF and AKT. These results indicates that the KIKK motif, assisting in the KRAS4A plasma membrane translocation, may mainly contribute to the differential effect of palmitoylation in the leukemogenesis of oncogenic KRAS4A and NRAS. These results also suggest that palmitoylation would be an effective target for both oncogenic KRAS4A and NRAS associated cancer therapy and that the KIKK motif should also be taken into account in the case of oncogenic KRAS4A associated cancer.

The KIKK motif has been recognized as a membrane-targeting motif [12, 23], but the mechanism was unknown. Electrostatical interaction of polybasic motifs with negatively charged lipids promotes protein binding with plasma membrane [33, 34]. But, the polybasic region of KRAS4B consists of a stretch of six lysine residuals. To test whether the KIKK motif target KRAS4A to membrane through electrostatical interaction or specific protein-protein interaction, we substituted all lysine residuals in the KIKK motif with arginine residuals and found no obvious functional change of oncogenic KRAS4A. In addition, substitution of lysine residuals in the KIKK motif with one, two, or three glutamine residuals results in its gradual decrease of transforming potential, suggesting that the KIKK motif of KRAS4A functions by providing electrostatic interaction with the plasma membrane through its positively charged amino acids.

Sequence analysis shows that both KRAS4A and NRAS harbor an RxKK (x: methionine or leucine) motif, which is designated as PB1 (polybasic domain 1) and was shown to promote the membrane localization [12, 23]. Our data show that mutating both the palmitoylation site and the KIKK motif like the effect of mutating the palmitoylation site in NRAS^G12D^, completely abolishes KRAS4A^G12D^’s leukemogenic potential. These data suggest that the RxKK motif is a much weaker membrane-targeting motif comparing to palmitoylation and KIKK. In addition to the KIKK motif and the RxKK motif, amino acids between 172 and 181 from KRAS4A increases the transforming activity of NRAS^G12D^ in NIH3T3, but not Ba/F3, cells (Fig. 4c, e), indicating that this region in KRAS4A may also help its plasma membrane localization.

The fraction of KRAS4A^G12D^, even the QIQQ mutant KRAS4A^G12D^, localized on the plasma membrane is significantly more than that of NRAS^G12D^ (Fig. 5 and data not shown). It is possible that palmitoylation of KRAS4A is more efficient than that of NRAS. It is noticeable that the amino acid sequence around the palmitoylation site is different between NRAS and KRAS4A, raising the possibility that palmitoylation of NRAS and KRAS4A may be catalyzed by different palmitoylacyltransferases. Given its implication in developing targeted cancer therapies, this possibility should be tested in the future.

Recurrent mutations of slicing factor genes have been found in hematological malignances, as well as some solid tumors, indicating aberrant splicing is involved in the pathogenesis of cancer [35–38]. Our data show that KRAS4A is expressed in all hematological malignant cell lines tested and that some cell lines harbor a particularly high level of KRAS4A (Fig. 1a). It is possible that deregulated splicing facilitates the expression of the *KRAS4A* splice variant in certain hematological malignant cells. Understanding the mechanism by which the *KRAS4A/4B* splicing is regulated might help to reveal alternative strategies for cancer therapy.

## Conclusions

Palmitoylation palys a critical role in KRAS4A leukemogenesis, but it is not essential as that in case of NRAS. The KIKK motif in KRAS4A functions as an additional membrane-targeting motif and makes a contribution to leukemogenesis by oncogenic KRAS4A. The studies suggest that therapies targeting KRAS4A palmitoylation might be effective in treating related malignancies and that interfering the KIKK membrane-targeting motif would enhance the therapeutic effectiveness.

## Methods

### DNA constructs

NRAS^G12D^- and NRAS^G12D/C181S^-containing MSCV-GFP-IRES (MGI) retroviral constructs were generated as previously described [17]. Wild-type *KRAS4A* human cDNA (NCBI accession number: NM_033360.3) was cloned into the MGI vector with N-terminal Myc-tag. *KRAS4A*^*G12D*^ (replacing the glycine residual at codon 12 with aspartic acid), *KRAS4A*^*G12D/C180S*^, *KRAS4A*^*G12D/QIQQ*^, *KRAS4A*^*G12D/C180S/QIQQ*^, *KRAS4A*^*G12D/RIRR*^, *KRAS4A*^*G12D/C180S/RIRR*^, *KRAS4A*^*G12D/C180S/QIKK*^, *KRAS4A*^*G12D/C180S/KIQK*^, *KRAS4A*^*G12D/C180S/KIKQ*^, *KRAS4A*^*G12D/C180S/QIQK*^, *KRAS4A*^*G12D/C180S/QIKQ*^, and *KRAS4A*^*G12D/C180S/KIQQ*^ mutants were generated using Quickchange Site-Directed Mutagenesis Kit (Stratagene, #200518). Genes containing fragment-swapped mutants NK, KN, NKN, KNK, NNK, and KKN were synthesized by GENEWIZ, followed by subcloning them into the MGI vector. Retroviral vectors expressing KRAS4A^G12D^, KRAS4A^G12D/C180S^, KRAS4A^G12D/QIQQ^, and KRAS4A^G12D/C180S/QIQQ^ mutants as N-terminal GFP-fusion proteins were created by cloning these mutational genes into EGFP-C1 plasmids, followed by subcloning them into the MSCV-IRES (without GFP) vector to generate MSCV-GFP/RAS mutants-IRES plasmids. Genes containing fragment-switching mutants NK, KN, NKN, KNK, NNK, and KKN were synthesized by GENEWIZ, followed by subcloning them into the MGI vector. All constructs were confirmed by DNA sequencing before use.

### RT-qPCR

Bone marrow total RNA of three AML patient samples with oncogenic KRAS mutations were collected from the centers of Shanghai Institute of Hematology (SIH). The primers for *KRAS4A* and *KRAS4B* were designed as previously described [23]. Primer sequences are as follows: for *KRAS4A*, forward: 5′-agacacaaaacaggctcagga-3′, reverse: 5′-ttcacacagccaggagtcttt-3′; for *KRAS4B*, forward: 5′-gactggggagggctttcttt- 3′, reverse: 5′-gcatcatcaacaccctgtct-3′. Results were normalized to a standard curve built by a dilution of MGI-KRAS4A and MGI-KRAS4B plasmid DNA.

The study was approved by the ethics committees of all participating centers. All patients were given informed consent for cryopreservation of bone marrow (BM) according to the Declaration of Helsinki.

### Retrovirus production and tittering

Retroviruses were produced with the constructs described above by individually cotransfecting them with the Pcl-Eco retrovirus packaging vector into the Bosc23 retroviral packaging cell line using a calcium phosphate precipitation method as previously described [25, 26]. Retroviral supernatant was harvested 48 h after transfection and tittered as previously described [25, 26].

### Bone marrow transduction/transplantation

Retroviral transduction and BM transplantation experiments were performed as previously described [26]. Briefly, BM cells were isolated from the 6–8-week-old donor mice pretreated with 5-fluorouracil (250 mg/kg). BM cells were infected with retroviruses each day for 2 days in transplant medium, followed by injecting 4 × 10^5^ cells into the tail vein of each lethally irradiated (2 × 3.8 Gy, 4 h between each dose) female recipient BALB/c mice. Retroviral titers were matched to 2 × 10^6^ TFUs before BM infection. Recipient mice were monitored weekly for signs of disease beginning on day 14 after transplantation. All the animal experiments were approved by The Animal Care and Welfare Committee of Shanghai Jiao Tong University School of Medicine.

### Hematological and immunophenotypic analysis

Peripheral blood (PB) was obtained from mice by tail bleeding and analyzed by using a pocH-100iV Diff hematology analyzer (Sysmex Corporation). After lysing the RBC, PB, bone marrow, spleen, or thymus cells were stained with conjugated antibodies to specific surface antigens. Mac-1, Gr-1, CD19, B220, CD3, CD4, and CD8 were used separately or in combination for immunophenotypic analysis of hematopoietic or leukemic cells (BD Pharmingen). All flow cytometric analyses were performed on an LSR II system (BD Biosciences). Data were analyzed with FlowJo software (Tree Star Inc.).

### Cell culture

Bosc23 cells were grown in Dulbecco’s Modified Eagle’s Medium (DMEM), 10 % Fetal bovine serum (GIBCO, Catalog 10099-141), and 1× penicillin/streptomycin. NIH3T3 cells were cultured in DMEM, 10 % bovine serum (GIBCO, catalog #16170078), and 1× penicillin/streptomycin. Ba/F3 cells were maintained in 1640 medium supplemented with 10 % fetal bovine serum (FBS), 1× penicillin/streptomycin, and 10 % IL-3 containing WEHI-3B conditional medium. NIH3T3 and Ba/F3 cell lines stably expressing KRAS4A^G12D^ and mutant KRAS4A^G12D^ genes were generated by retroviral transduction as described [17, 26]. All cell lines were sorted by FACS to >95 % GFP positive homogeneity.

### Cell proliferation assay and microscopic analysis

After sorting by FACS, Ba/F3 cells transduced with KRAS4A^G12D^ or mutant KRAS4A^G12D^ genes expressing retroviruses were washed with 1640 twice to remove IL-3 and resuspended with 1640 containing 10 % FBS and 1× penicillin/streptomycin. Cells were plated in triplicate into 6-cm dishes in the absence of IL-3 and were stained with trypan blue to exclude nonviable cells and counted manually under a light microscope every 3 days until the cell lines fully grew or died. Student *t* test was used for the comparison of the cell numbers in each time point. NIH3T3 cells expressing KRAS4A^G12D^ or mutant KRAS4A^G12D^ genes formed tight spheres after culturing for 6 days and were photographed using an Olympus digital camera.

### Soft agar colony forming assay

To generate 0.6 % soft agar, 1.5 ml 4 % agarose with 8.5-ml cell culture media (Dulbecco’s modified Eagle’s medium + 20 % bovine serum + 1.2 times Pen-Strep) was mixed. A total of 2 mL of 0.6 % bottom agar (incubated in 42 °C) was then platted into each well of a 6-well tissue culture plate. FACS-purified NIH3T3 cell lines expressing MGI vector or RAS mutants were diluted to 2 × 10^5^ cells/ml, 2 × 10^4^ cells/ml, or 2 × 10^3^ cells/ml in. Triplicate 3-mL cells suspended in 0.3 % soft agar (cell dilution + 0.6 % soft agar with 1:1 ratio) were added on top of the jellified bottom agar for each type of transduced cells and incubated in CO_2_ incubator at 37 °C. Colonies were counted under a light microscope at day 14 after plating.

### Western blot analysis

Western blot analyses were performed as previously described [17, 26, 39]. Briefly, 90 % confluent NIH3T3 cells were serum-starved for 22 h. Cells were counted and lysed in 1× SDS running buffer, heated at 100 °C for 10 min, and centrifuged to remove debris. Lysates were resolved on 10 % polyacrylamide gels, transferred to nitrocellulose membranes, and blotted with the following primary antibodies: anti-KRAS4A specific antibody (1:500; Santa Cruz Biotechnology, sc-522), anti-RAS antibody (1:2000; Cell Signaling Technology, #3965), anti-actin (1:5000; Sigma-Aldrich, A1978), anti-GFP (1:2000; Cell Signaling Technology, #2555), and phospho-c-RAF, c-RAF, phoshpo-MEK1/2, MEK1/2, phospho-ERK1/2, ERK1/2, phospho-AKT, AKT, phospho-S6RP, and S6RP (All 1:2000; Cell Signaling Technology). HRP-labeled goat anti-mouse IgG or goat anti-rabbit IgG was used as a secondary antibody.

### Cellular localization analysis of RAS proteins

NIH3T3 cells expressing KRAS4A^G12D^ with GFP fused to its N terminus or expressing GFP-fusion versions of KRAS4A^G12D^ PTM or KIKK motif mutant proteins were grown overnight. Fluorescence was visualized and photographed on a Leica TCS 7300 Spectral Confocal Microscope. The images were analyzed by using LAS AF Lite Software (Leica). The plasma membrane localization ratios of KRAS4A mutants were counted by (pixel(plasma membrane)/pixel(sum)). Ten representative cells of each group were counted and analyzed. Student *t* test was used for statistical data analysis.

### Statistical analysis

Graphpad Prism 6 software was used for statistical data analysis. Student *t* test was used for mean comparison, whereas the Kaplan-Meier survival curve and log-rank test were used for survival analysis.
